# Ramipril Alleviates Oxaliplatin-Induced Acute Pain Syndrome in Mice

**DOI:** 10.3389/fphar.2021.712442

**Published:** 2021-07-19

**Authors:** Hichem Bouchenaki, Aurore Danigo, Amandine Bernard, Flavien Bessaguet, Laurence Richard, Franck Sturtz, David Balayssac, Laurent Magy, Claire Demiot

**Affiliations:** ^1^ EA 6309 - Myelin Maintenance and Peripheral Neuropathy, Faculties of Medicine and Pharmacy, University of Limoges, Limoges, France; ^2^ Department of Neurology, Reference Center for Rare Peripheral Neuropathies, University Hospital of Limoges, Limoges, France; ^3^ Neuro-Dol, UMR1107 INSERM, University of Clermont Auvergne, CHU Clermont-Ferrand, Clermont-Ferrand, France

**Keywords:** oxaliplatin, ramipril, cold allodynia, mechanical allodynia, neuropathic pain

## Abstract

Oxaliplatin is a key drug for colorectal cancer that causes OXP-induced peripheral neuropathy, a dose-limiting effect characterized by cold and tactile hyperesthesia. The relationship between the sensory nervous system and modulation of the renin-angiotensin system has been described, focusing on pain and neurodegeneration in several animal models. We assessed the effect of the RAS modulator, ramipril, an angiotensin converting-enzyme inhibitor in a mouse model of OXP-induced acute pain syndrome. OXP was administered in Swiss mice at a cumulative dose of 15 mg/kg (3 x 5 mg/kg/3 days, i.p.). RAM was administered i.p. every day from 24 h before the first OXP injection until the end of the experiments. We evaluated OIAS development and treatment effects by sensorimotor tests, intraepidermal nerve fiber and dorsal root ganglia-neuron immunohistochemical analyses, and sciatic nerve ultrastructural analysis. OXP-treated mice showed tactile allodynia and cold hypersensitivity, without motor impairment and evidence of nerve degeneration. RAM prevented cold sensitivity and improved recovery of normal tactile sensitivity in OXP-treated mice. Our finding that RAM alleviates OXP-induced pain is a step towards evaluating its therapeutic potential in patients receiving OXP treatment.

## Introduction

Oxaliplatin, a platinum-based agent mostly used to treat colorectal cancer, frequently induces peripheral neuropathy, decreasing patients’ quality of life (Selvy et al., 2020). Development of oxaliplatin-induced peripheral neuropathy often leads to lower doses or disrupt treatment, thus limiting treatment efficacy and decreasing survival rate (Gewandter et al., 2017). OXP induces chronic cumulative sensory neuropathy (Pachman et al., 2015), probably caused by an accumulation of platinum in the dorsal root ganglia (Sprowl et al., 2013). This is often preceded by a specific acute syndrome, appearing during or within hours after each infusion (Pasetto et al., 2006), mainly consisting of numbness, paresthesia, dysesthesia, and pain, with cold allodynia (Pachman et al., 2015). Although not all patients with an OXP-induced acute syndrome will develop a long term disabling chronic sensory neuropathy, this OIAS probably represents the first step towards long term neurodegeneration (Park et al., 2009). Therefore, examining how different drugs could counteract these acute symptoms might be meaningful to develop strategies to prevent chronic OIPN. Because no treatment can be recommended for the prevention of chemotherapy-induced peripheral neuropathies and only duloxetine is moderately recommended for its treatment (Jordan et al., 2020; Loprinzi et al., 2020), preventing and limiting its progression by an appropriate therapeutic approach is a key priority. The renin angiotensin system is known for its involvement in blood pressure regulation and ion homeostasis (Rettig et al., 1986). Briefly, angiotensin-converting enzyme converts angiotensin I to angiotensin II, which mainly interacts with Ang II type 1 receptor and Ang II type 2 receptor. Most of the RAS components are known to be expressed into the sensory nervous system, tending to demonstrate the presence of a local RAS in the peripheral nervous system (Bessaguet et al., 2016). Various preclinical studies have highlighted the involvement of RAS modulation by the use of ACE inhibitors or angiotensin receptor blockers, in neuroprotection and pain control (Gallinat et al., 1998; Lucius et al., 1998; Patil et al., 2010; Pavel et al., 2013; Kaur et al., 2015; Yuksel et al., 2015; Bessaguet et al., 2017; Danigo et al., 2021). Modulation of RAS was neuroprotective in rodent models of traumatic nerve injury like chronic constriction injury and sciatic nerve transection, as well as in rodent models of diabetic neuropathy or toxic neuropathy (CIPN) (Oltman et al., 2008; Kaur et al., 2015; Yuksel et al., 2015; Bessaguet et al., 2017; Kim et al., 2019). Previously, we and others showed that ARBs were able to restore normal sensitivity in models of vincristine- and paclitaxel-associated CIPN (Bessaguet et al., 2018; Kim et al., 2019). Antitumoral activity of both paclitaxel and vincristine are based on the disruption of microtubule dynamics, which may probably cause an impairment of axoplasmic transport leading to neuropathic disorders. Among the other “traditional” chemotherapies, platinum agents, as OXP, act differentially by forming platinum-DNA adduct, and therefore lead to a different neurotoxicity. Neuroprotective effects of RAS modulation on platinum compound-induced neurotoxicity have not yet been evaluated. Although, a retrospective observational study showed that patients with a long lasting ACEIs treatment seemed to be less affected by OIPN symptoms (Roldan et al., 2017; Uchida et al., 2018). Hence, we evaluated the preventive effect of ramipril in a new mouse model of OIAS. This model is characterized by functional changes without evidence of sensory nerve fiber degeneration at the peripheral nerve and DRG level.

## Materials and Methods

This study was conducted in accordance with the guidelines for the ethical care of experimental animals of the European Community (2010/63/EU) and was submitted to the French Ministry of Higher Education and Research and approved (number 11280#2017091510483336). Animal experiments are reported in compliance with ARRIVE guidelines (Percie du Sert et al., 2020). All effort was made to limit suffering and the number of animals used in the following experiments.

### Animals

Swiss male mice (6–7 weeks old, 25–30 g) from Janvier labs (France) were housed in plastic cages and maintained on a 12 h light/dark cycle with food and water available ad libitum (BISCEm-animal care and facility center). Mice were randomly assigned to OXP or its vehicle (control group: CTRL), thereafter mice were randomly assigned to ramipril treatment or its vehicle, defining the following four treatment groups: control-vehicle (CTRL-VEH), oxaliplatin-vehicle (OXP-VEH), control-ramipril (CTRL-RAM) and oxaliplatin-ramipril (OXP-RAM).

**FIGURE 1:**
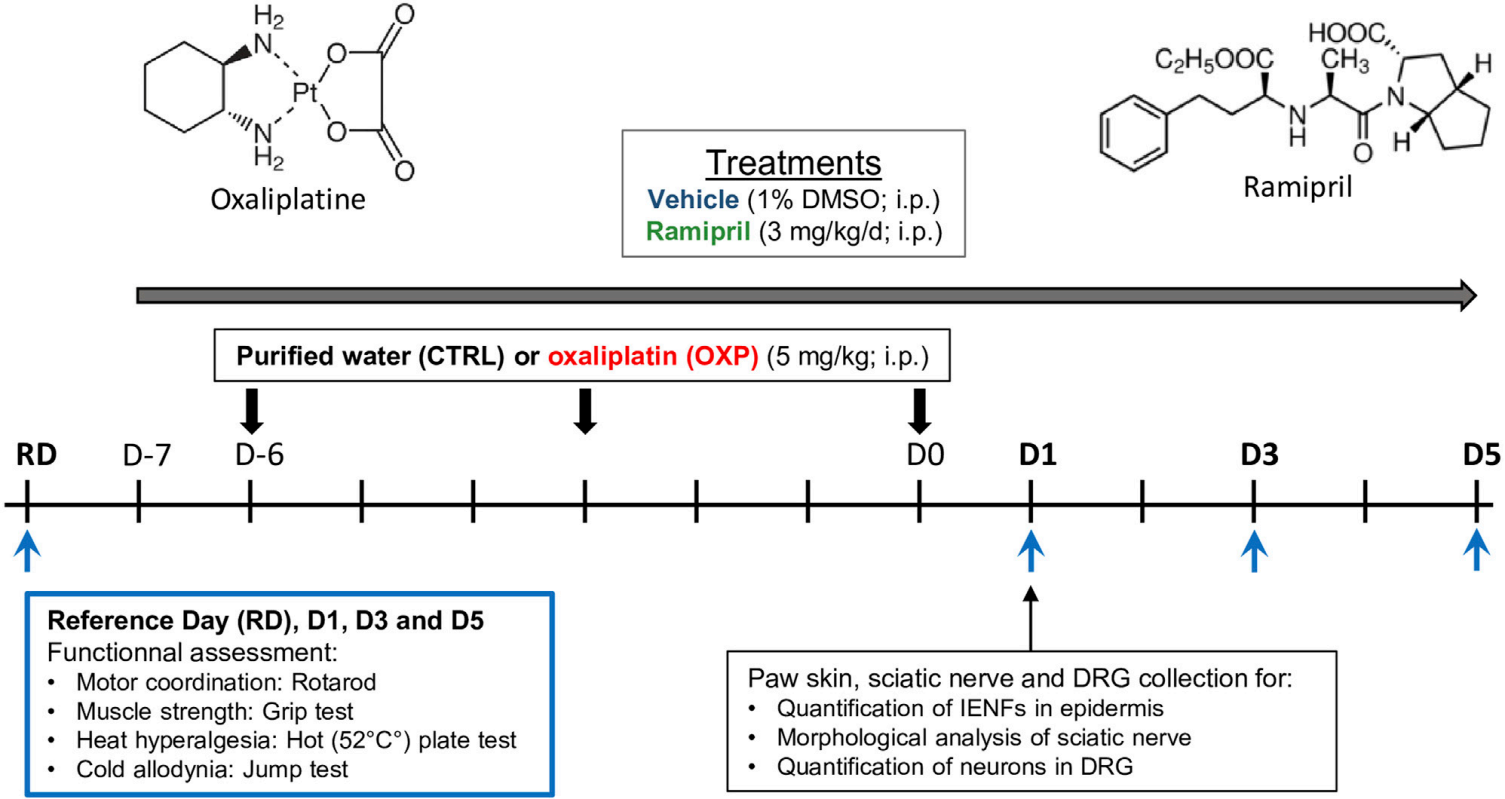
Schematic representation of the study design. CTRL, control; D, day; DMSO, diméthylsulfoxyde, DRG, dorsal root ganglion; IENFs, intraepidermal nerve fibers; i.p., intraperitoneal; RD, reference day.

Behavioral tests were performed on day 1 (D1), D3, and D5 following the last OXP injection, except for the jump test, which was performed on D1 and D3 only to avoid acclimation of the mice to the test. All animals were submitted to the functional tests, except for the jump test, the day before the start of experiment (reference day: RD) to obtain the baseline for each animal. Immunohistochemistry and morphological analyses were performed one day after the last OXP injection, the time corresponding to the maximum of sensory impairment (Figure 1).

### Treatments

CIPN was induced by three injections of OXP (1 injection every 3 days) (5 mg/kg/3 days, intraperitoneally [i.p.]; Hospira, France). Control mice received an equivalent volume of the OXP diluent (purified water, i.p.). Treatments with ramipril (3 mg/kg/day, i.p.; Sigma, France) were started one day before the first OXP injection and administered each day for the next 10 days (Figure 1). The dose of 3 mg/kg/i.p. of ramipril in mice is equivalent to a dose of 30 mg/day *per os* in human, corresponding to a high dose (Alhusban et al., 2014). Although, high dose of ramipril had already shown neuroprotective effect in a rodent model of peripheral neuropathy (Kaur et al., 2015). Ramipril was diluted in a final vehicle solution of 1% dimethyl sulfoxide (DMSO) in saline solution (0.9% NaCl). VEH mice received injections of an equivalent volume of the vehicle used for ramipril. The volume of i.p injections was 100 µl/10 g of body weight.

### Behavioral Tests

#### Motor Coordination

Motor coordination was assessed using the rotarod test (Bioseb, France) (Callizot et al., 2008). Three days before the start of the experiment (RD), mice were trained to walk against the motion of a rotating rod at a speed of 4 rpm. On the days of test, mice were placed on the rotarod and gradual 1 rpm/s acceleration was applied for 30 s, after which the speed was constant (4 rpm/s). The holding time (seconds) was recorded. The cut-off time was set on 60 s. Each test session consisted of three trials, separated by 5 min, and the mean value was calculated.

#### Muscle Strength

Muscle function was assessed with a grip strength meter (Bioseb, France) (Caillaud et al., 2020). Mice were held by the tail over the mesh of the meter, and once the forepaws, but neither hind paw, were both firmly grasping the grid, the mice were pulled along the axis of the force sensor until they were unable to retain their grip. The grip strength (grams) was recorded. Each test consisted of three trials, separated by 5 min, and the mean value was calculated.

#### Heat Hyperalgesia

Thermal nociception was assessed using the hot-plate test (Bioseb, France) (Bessaguet et al., 2017). Mice were placed on a 52°C hotplate for less than 30 s to avoid potential tissue damage. The latency (s) before first withdrawal criterion was recorded. The criteria of withdrawal included shaking, licking, or jumping on the hot plate. Mean latency was expressed as the threshold of an individual animal to thermal stimulation. Each test session consisted of three records separated by 5 min.

#### Tactile Allodynia

Tactile allodynia was assessed using von Frey filaments (Bioseb, France). Mice were placed in a plastic cage with a wire mesh floor which allowed access to the paws. The plastic cage was covered with an opacity cup to avoid visual stimulation. The area tested was the mid-plantar left hind paw. Mechanical threshold was tested using a modification of the simplified up-down method (Bonin et al., 2014). A test round started with filament #6 (0.40 g) and progressed to higher or lower filament value depending on the animal’s response. Each animal went through three test rounds for each paw at each experimental condition. Mechanical threshold is expressed as percentage according to baseline (%).

#### Cold Hypersensitivity

Thermal nociception was assessed using the jump to escape test (Descoeur et al., 2011). Noxious cold tolerance was assessed using a dynamic cold plate (Bioseb, France). Animals were placed on the test arena with the floor temperature progressively cooled from 22 to 3°C at a rate of 5°C/min. This procedure allows the paw surfaces to be cooled at the same rate as the floor arena. Nocifensive behavior (jumps) was noted as a function of cooling. Thus, we obtained a curve representing the number of jumps according to temperature for each mouse. Then, the area under the curve (AUC) was calculated for each animal and pooled in a histogram. Statistical analyses were performed on the AUC.

### Immunohistochemistry of Footpad Skin and Dorsal Root Ganglia Neurons

To assess sensory innervation, animals (*n* = 6) were euthanized by transcardiac perfusion with phosphate buffer saline (PBS), followed by buffered 4% paraformaldehyde (PFA) solution. Then, footpads were removed by punch biopsy (diameter of 3 mm), post-fixed overnight in 4% PFA, cryoprotected (30% sucrose) and frozen at −20°C. Sections were cut on a cryostat set to 20 mm and incubated overnight at room temperature with primary antibody to protein gene product 9.5 (PGP9.5, 1:50, Abcam, Paris, France). Sections were then incubated with appropriate secondary antibody AF594-conjugated (1:500; Life Technologies, Saint-Aubin, France) during 2 h at room temperature. Epidermal nerve fibers were blindly counted under 400× magnification (Eclipse 50i, Nikon Instruments), according to established guidelines for Human (Lauria et al., 2005). The length of the dermo-epidermal junction was determined with NIS-Elements BR 2.30 software (Nikon) and was defined as the epidermal length. Epidermal nerve density was defined as the number of intact epidermal nerves crossing the dermo-epidermal junction divided by the epidermal length. Three slides per mouse were counted.

Two lumbar (L4 and L5) DRG per mouse were collected to assess the DRG neuron density by counting neuron cellular bodies visualized by PGP9.5, calcitonin gene-related peptide (CGRP), or substance P (SP) immunohistochemistry (Table 1). Each DRG section was systematically photographed at 200x under a fluorescence microscope (Eclipse 50i; Nikon Instruments, France). Immunoreactive DRG neurons were counted and the area containing neurons measured using NIS-Elements BR2.30 software (Nikon). The density of neurons positive for PGP9.5 is expressed as neurons/mm2. The density of peptidergic neurons was expressed as CGRP^+^ or SP^+^ neurons/PGP 9.5^+^ neurons. Three sections per DRG were counted.

**TABLE 1:** Effects of OXP on motor performance and the heat nociceptive response.

Group	CTRL-VEH				OXP-VEH			
Days	RD	D1	D3	D5	RD	D1	D3	D5
Rotarod test (holding time (s))	15.42 ± 2.1	18.33 ± 5.8	19.50 ± 6.5	22.59 ± 6.8	15.28 ± 1.7	20.63 ± 4.7	20.14 ± 4.1	20.84 ± 4.8
Grip strength (g)	183.9 ± 8.51	174.9 ± 5.33	179.0 ± 4.8	200.1 ± 5.7	195.2 ± 10	162.6 ± 6.4	189.6 ± 8.5	196.3 ± 6.8
Hot plate test (withdrawal latency (s))	14.35 ± 1.1	15.47 ± 1.21	19.86 ± 1.41	17.57 ± 1.1	14.56 ± 1.3	17.57 ± 0.9	17.46 ± 1.6	18.88 ± 0.6

OXP-treated mice received OXP at a cumulative dose of 15 mg/kg (5 mg/kg/3 days, intraperitoneal). *n* = 10 mice per group. g, grams; RD, reference day; OXP, oxaliplatin; s, seconds; VEH, vehicle.

### Morphological Analysis of Sciatic Nerves

To assess the presence and morphology of unmyelinated nerve fibers, sciatic nerves were dissected after transcardiac perfusion with 2.5% glutaraldehyde diluted in Sorensen buffer, dehydrated, and embedded in Epon 812 resin (Euromedex, France). Semi-thin sections were stained with toluidine blue. Ultrathin sections were stained with uranyl acetate and lead citrate and observed under an electron microscope (Jeol 1011, Jeol, United States). Four images per animal (*n* = 3/group) were captured at 3000x magnification and the number of myelinated fibers per mm^2^ counted to calculate the density.

### Data Analysis

Data were analyzed using GraphPad Prism 8 and expressed as the mean ± standard error of the mean (SEM). When statistical significance was identified by mixed-effects model statistical methods, individual comparisons were subsequently tested by Tukey’s multiple comparison test. Degree of significance was represented as follow: *p-value < 0.05, **p < 0.01, ***p < 0.001, ****p < 0.0001.

## Results

### Characterization of Oxaliplatin-Induced Acute Pain Syndrome

#### Short Course of Oxaliplatin Does Not Affect Motor Performance or Heat Sensitivity

The motor coordination of CTRL-VEH and OXP-VEH mice was evaluated using the rotarod test. There was no difference in the holding-time between OXP-VEH and CTRL-VEH mice on D1, D3, or D5 (Table 1). Muscular strength was assessed using the grip test. There was also no difference in the grip strength between OXP-VEH and CTRL-VEH mice on D1, D3, or D5 (Table 1). Thermal nociception, evaluated with the 52°C hotplate test, was not affected by OXP treatment on D1, D3, or D5 (Table 1).

#### Short Course of Oxaliplatin Does Not Affect the Morphology of Sensory Nerve Fibers

**FIGURE 2:**
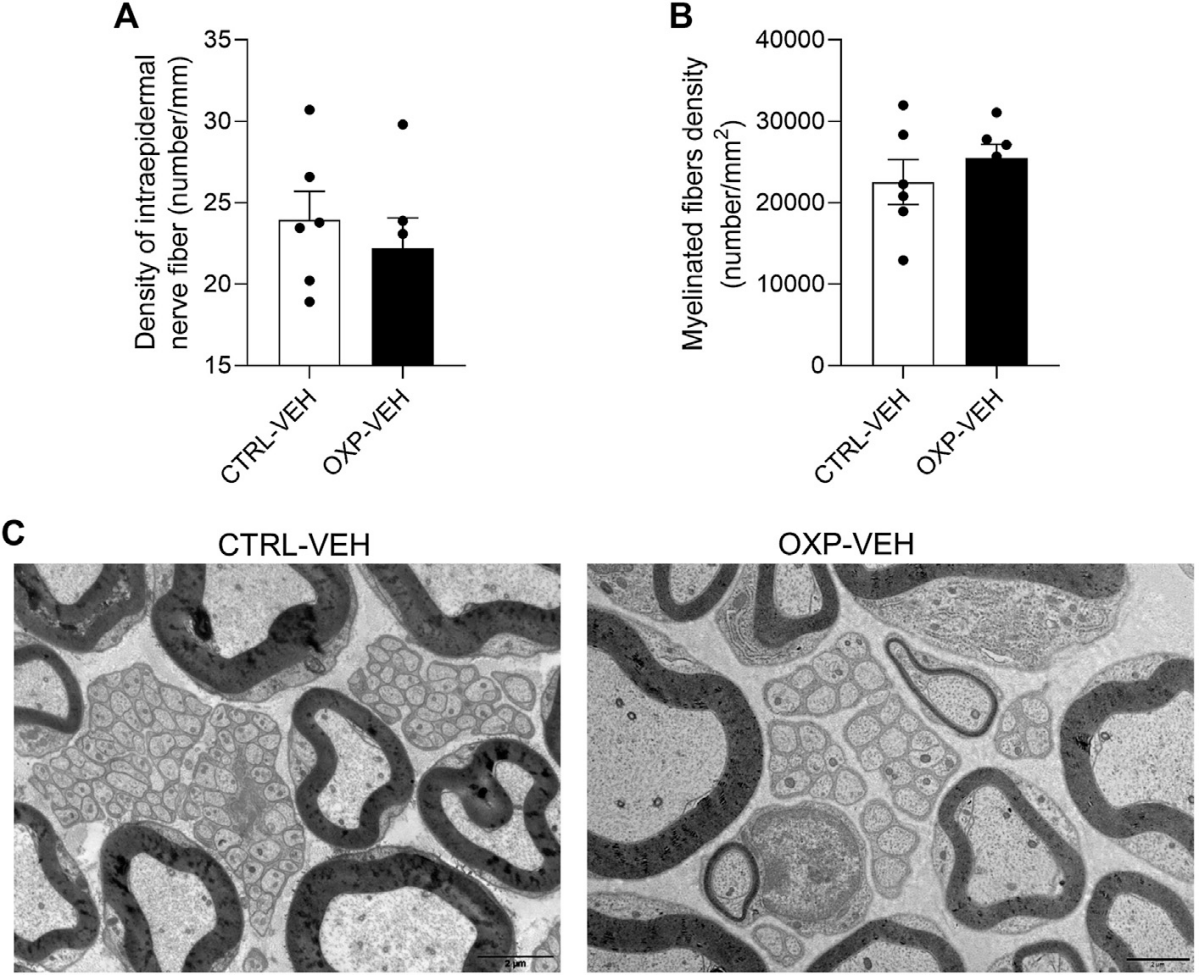
Effect of OXP on peripheral nerve fiber morphology. Footpad skin of CTRL-VEH and OXP-VEH mice were immunostained with PGP9.5 **(A)** Quantification of IENF positive for PGP9.5. Sciatic nerve fibers were examined by electron microscopy. **(B)** Quantification of myelinated fiber density. Analysis of three nerves per group and four fields of view per nerve **(C)** Visualization of myelinated and unmyelinated nerve fibers in the sciatic nerve. Scale bar = 2 µm *n* = 6 mice per group. OXP-treated mice received OXP at a cumulative dose of 15 mg/kg (5 mg/kg/3 days, intraperitoneal). CTRL: control, DRG: dorsal root ganglion, IENF: intraepidermal nerve fiber, PGP9.5: protein gene product 9.5, OXP: oxaliplatin, VEH: vehicle.

**FIGURE 3:**
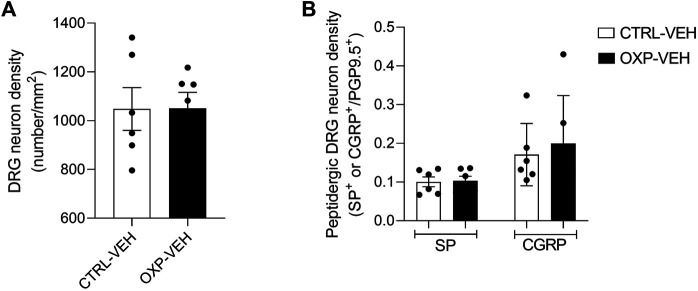
Effect of OXP on dorsal root ganglion neurons. DRG of CTRL-VEH and OXP-VEH mice were immunostained with PGP9.5 and substance P (SP) or calcitonin-gene related peptide (CGRP). **(A)** Quantification of PGP9.5^+^ total DRG neuron density. **(B)** The density of peptidergic neurons is expressed as SP^+^ or CGRP^+^ neurons/PGP9.5^+^ neurons. OXP-treated mice received OXP at a cumulative dose of 15 mg/kg (5 mg/kg/3 days, intraperitoneal). CTRL: control, DRG: dorsal root ganglion, PGP9.5: protein gene product 9.5, OXP: oxaliplatin, VEH: vehicle.

We quantified and examined morphological aspect of sensory nerve fibers at the cell body (DRG) and the terminal level (IENF). There was no significant difference in the densities of IENF (*p* = 0.5111, Figure 2A) and DRG neurons (*p* = 0.9787, Figure 3A) between OXP-VEH and CTRL-VEH mice. There was no visible morphological effect of OXP on DRG neurons under our conditions. Moreover, we quantified the SP^+^ and CGRP^+^ DRG neurons to explore the effect of OXP on neuropeptide depletion. There was no visible effect of OXP on peptidergic neuron population under our conditions (Figure 3B).

#### Short Course of Oxaliplatin Does Not Affect the Morphology of Sciatic Nerve Fibers

OXP did not affect the morphology nor density of myelinated nerve fibers in sciatic nerves (Figures 2B,C). There were also no noticeable changes in unmyelinated fiber morphology in OXP-VEH mice relative to CTRL-VEH mice (Figure 2C).

#### Short Course of Oxaliplatin Induces Significant Tactile and Cold Allodynia

**FIGURE 4:**
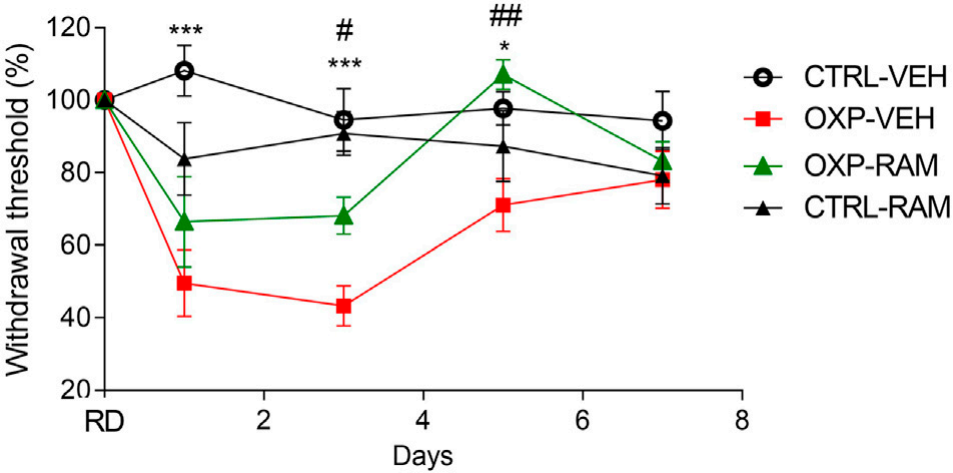
Effects of ramipril on OXP-induced mechanical allodynia. Results are compared using mixed-effects model test followed by Tukey’s multiple comparisons test (Factor treatment: F_3.044, 102_ = 17.79, *p* < 0.0001, time: F_3, 36_ = 15.73, *p* < 0.0001 and interaction: F_12, 134_ = 4.653, *p* < 0.0001). Each group represents the mean of 8–10 animals, and the error bar indicates the SEM. ***p < 0.001, *p < 0.05 OXP-VEH *vs.* CTRL-VEH groups. ^##^ *p* < 0.01, ^#^ *p* < 0.05 OXP-VEH *vs.* OXP-RAM groups. *n* = 10 per group. OXP-treated mice received OXP at a cumulative dose of 15 mg/kg (5 mg/kg/3 days, intraperitoneal). RAM-treated mice received daily injection of ramipril at the dose of 3 mg/kg (intraperitoneal). OXP: oxaliplatin, RAM: ramipril, RD: reference day, VEH: vehicle.

There was no difference in tactile sensitivity between any groups on the reference day. OXP-VEH mice showed significant tactile allodynia compared to CTRL-VEH mice from D1 to D5 (D1: *p* = 0.0003, D3: *p* = 0.0004, D5: *p* = 0.0275, OXP-VEH *vs.* CTRL-VEH). Mechanical threshold returned to baseline 7 days after the last OXP injection (OXP-VEH *vs*. CTRL-VEH, *p* = 0.4980) (Figure 4).

**FIGURE 5:**
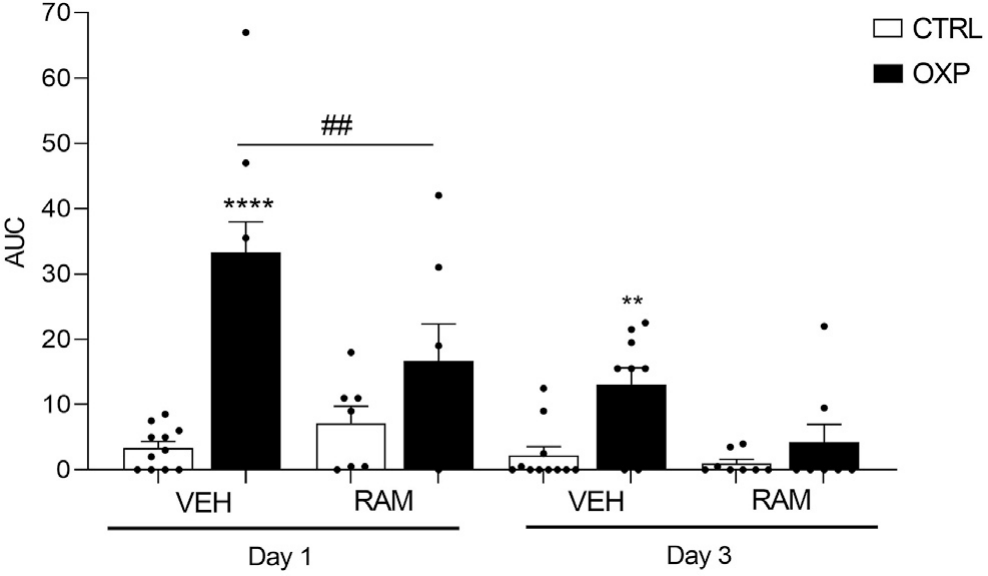
Effects of ramipril on OXP-induced cold hypersensitivity. Results are compared using mixed-effects model test followed by Tukey’s multiple comparisons test (Factor treatment: F_5, 47_ = 15.57, *p* < 0.0001, time: F_1, 45_ = 38.68, *p* < 0.0001 and interaction: F_5, 45_ = 4.621, *p* = 0.0017). Each group represents the mean of 8–10 animals, and the error bar indicates the SEM. ****p < 0.001, **p < 0.01 OXP-VEH *vs.* CTRL-VEH groups. ^##^ *p* < 0.01 OXP-VEH *vs.* OXP-RAM. *n* = 10 per group. OXP-treated mice received OXP at a cumulative dose of 15 mg/kg (5 mg/kg/3 days, intraperitoneal). RAM-treated mice received daily injection of ramipril at the dose of 3 mg/kg (intraperitoneal). AUC: area under the curve, OXP: oxaliplatin, RAM: ramipril, VEH: vehicle.

OXP-treated mice showed cold induced allodynia on D1 (OXP-VEH *vs*. CTRL-VEH, *p* < 0.0001) and D3 (OXP-VEH *vs*. CTRL-VEH, *p* = 0.0134), as the AUC (representing the number of jumps according to temperature) of CTRL-OXP-VEH mice was higher than that of CTRL-VEH mice (Figure 5).

### Effect of Ramipril On Oxaliplatin-Induced Pain

#### Ramipril Alleviates Oxaliplatin-Induced Tactile Allodynia

Ramipril had no effect on mechanical responses of the control groups on D1 (CTRL-RAM *vs.* CTRL-VEH, *p* = 0.1527), D3 (CTRL-RAM *vs.* CTRL-VEH, *p* = 0.9318), or D5 (CTRL-RAM *vs.* CTRL-VEH, *p* = 0.6117) (Figure 3). Ramipril tended to improve tactile sensitivity in OXP-treated mice at D3 (OXP-RAM *vs.* OXP-VEH, *p* = 0.0396) and significantly restored normal tactile sensitivity at D5 (OXP-RAM *vs.* OXP-VEH, *p* = 0.0055 and *vs*. CTRL-VEH, *p* = 0.6610) (Figure 4).

#### Ramipril Restores Cold Sensitivity

Ramipril did not influence cold responses in the control group on D1 (CTRL-RAM *vs.* CTRL-VEH, *p* = 0.5265) and D3 (CTRL-RAM *vs.* CTRL-VEH, *p* = 0.9908) (Figure 4). Ramipril significantly improved cold sensitivity on D1 in OXP mice (OXP-RAM *vs.* OXP-VEH, *p* = 0.0014) and restored normal cold sensitivity on D3 (OXP-RAM *vs.* CTRL-VEH, *p* = 0.9953) (Figure 5).

## Discussion

Our main findings were: 1) the development and characterization of a mouse OIAS model, without evidence of nerve degeneration, and 2) ramipril alleviates cold allodynia and improves mechanical sensitivity in OXP-treated mice.

Mice treated with short course of OXP did not develop abnormal motor coordination or muscular strength behaviors, suggesting that proprioceptive and motor Aα-fibers were not altered by OXP injections. This was confirmed by electron microscopy. The absence of motor deficit is consistent with observations made in other mouse strains; C57BL6J and BALB/c adult male mice (Ta et al., 2009; Andoh et al., 2011; Descoeur et al., 2011; Nassini et al., 2011; Renn et al., 2011; Zhao et al., 2012; Azevedo et al., 2013; Toyama et al., 2014; Zhao et al., 2014). The hot plate test showed no alteration of heat sensitivity, as previously shown in mouse models (Ta et al., 2009; Renn et al., 2011; Toyama et al., 2014). Immunohistochemistry analysis and sciatic nerve electron microscopic examination showed no axonal degeneration in the DRG, neuropeptide depletion, loss of IENFs, or nerve degeneration, meaning our model mimics acute neurotoxicity, without leading to long term degeneration. Previous studies on various experimental models showed that cisplatin or OXP do not induce axonal degeneration, consistent with our findings (Barajon et al., 1996; Jamieson et al., 2005). However, IENF loss has been previously described with different experimental conditions (Boyette-Davis and Dougherty, 2011; Xiao et al., 2012; Toyama et al., 2014). Nevertheless, in these studies, OXP was administered for several weeks, inducing neuropathic symptoms for a longer period than in our study. Moreover, loss of IENFs cannot explain pain in all cases, suggesting that different mechanism underpin the genesis of pain during anticancer therapy (Koskinen et al., 2011).

The relevance of our OIAS model resides in its being transient. Indeed, mice developed transient cold hypersensitivity, which consistently reproduced OXP-induced acute symptoms in humans, typically appearing with the second or third cycle of treatment during the infusion or within one or two days of OXP administration and disappearing within a few days (Staff et al., 2017). Clinically, cold-induced symptoms are the most prominent and disabling manifestations of OXP in the short term (Argyriou et al., 2008). Our OXP mice also develop transient tactile allodynia, highlighted by the von Frey test. Tactile allodynia has been previously described in patients as a symptom of OXP neurotoxicity (Binder et al., 2007) and widely described in rodent models (Hopkins et al., 2016).

Molecular basis of OIAS was not fully elucidated, although several interesting tracks have been explored in the literature. Among them, studies suggested that OXP induces a hyperexcitability of primary sensory neurons related to a dysregulation or variation of the expression of ion channels involved in mechanical and cold sensitivity (Grolleau et al., 2001; Park et al., 2009; Park et al., 2011; Kawashiri et al., 2012). More particularly, others recently reported that OIAS was associated with a downregulation of K^+^ channels in the DRG, following a single dose of OXP (Pereira et al., 2021). Another mechanism of OIAS is the generation of oxidative stress at the spinal level. The resulting reactive oxygen species (ROS) would induce a neuroinflammation, at the origin of hypersensitization of primary sensory neurons (Di Cesare Mannelli et al., 2012; Zhang et al., 2020; Agnes et al., 2021).

In summary, under our experimental conditions, three injections of OXP at 5 mg/kg in Swiss mice induced OIAS, characterized by transient tactile allodynia and cold hypersensitivity, associated with no impairment in motor performance or heat nociception, without overt nerve degeneration.

Ramipril, an ACE inhibitor, decreases Ang II synthesis and so AT1R/AT2R stimulation (Weber, 1999). Here we showed that ramipril improves tactile sensitivity and prevents cold sensitivity. The effect of ramipril could be mediated by the increase activity of ACE2 which converts angiotensinogen to Ang (1–7) producing antinociceptive action *via* Mas receptor (Forte et al., 2016; Ogata et al., 2019). Moreover, it was demonstrated that antinociceptive effect of Ang (1–7) involved K^+^ channels (Costa et al., 2014). Ramipril was previously shown to improve neuropathic pain in a chronic constriction-injury mouse model due to its antioxidant properties, following the decrease in Ang II levels (Kaur et al., 2015). In these study, ramipril treatment improves effects of nerve injury-induced increase in thiobarbituric acid substances (TBARS) and decrease in glutathione levels. Moreover, blockade of ACE prevents the formation of Ang II but also avoid the degradation of kinins and downregulates the bradykinin B1 receptor (Estrela et al., 2020). B1 receptor is upregulated during neuroinflammation and is involved in neuropathic pain (Ferreira et al., 2005; Cernit et al., 2020). Thus, downregulation of B1 receptor could be involved in the beneficial effect of ramipril. As oxidative stress and neuroinflammation have been shown to contribute to OIPN, they could contribute to the antinociceptive effect of ramipril.

In our study, beneficial effect of ramipril seemed to be unequal between cold and tactile pain. Tactile and cold sensitivity are transmitted by various types of fibers. As Aβ fibers participate in touch *i.e.* a non-painful stimulus, they are involved in the development of tactile allodynia, a non-nociceptive sensation. C and Aδ fibers are responsible for noxious and non-noxious perception of cold. Thus, the simplest explanation for such a difference in the effect of ramipril between tactile and cold allodynia is that these manifestations are linked to different fiber/DRG neuron subpopulations.

To conclude, we have shown that high dose of ramipril improves the recovery of cold sensitivity and alleviates OXP-induced tactile allodynia. Further investigation using a dose-response protocol to demonstrate whether a decrease in the dose of ramipril correlates with a decrease or not in the anti-allodynic effect in this model would be needed. As ramipril is a relatively inexpensive drug which has been demonstrated to be safe and well tolerated, our results favor clinical evaluation of the preventive therapeutic potential of ramipril in OXP-treated patients. Retrospective observational studies showed that ACEI administration in cancer patients under anticancer therapy is well tolerated (Roldan et al., 2017; Uchida et al., 2018). Moreover, the same studies showed that long lasting treatment with ACEI/ARB seems to protect sensory myelinated nerve fiber function from chemotherapy-induced neurotoxicity (Roldan et al., 2017). Thus, demonstrating the preventive effect of this drug on the development of chronic CIPN, would be the next step towards evaluation of the preventive therapeutic potential of ramipril in patients receiving OXP-based chemotherapy.

## Data Availability

The original contributions presented in the study are included in the article/Supplementary Material, further inquiries can be directed to the corresponding author.

## Abbreviation

CIPN, chemotherapy-induced peripheral neuropathy; OIAS, oxaliplatin-induced acute pain syndrome; OIPN, oxaliplatin-induced peripheral neuropathy; OXP, oxaliplatin; RAM, Ramipril; RAS, renin angiotensin system.
